# Towards an Early Warning System for Rhodesian Sleeping Sickness in Savannah Areas: Man-Like Traps for Tsetse Flies

**DOI:** 10.1371/journal.pntd.0001978

**Published:** 2012-12-27

**Authors:** Glyn A. Vale, David R. Hall, Andrew Chamisa, Stephen J. Torr

**Affiliations:** 1 Natural Resources Institute, University of Greenwich, Chatham, United Kingdom; 2 Southern African Centre for Epidemiological Modelling and Analysis, University of Stellenbosch, Stellenbosch, South Africa; 3 Division of Tsetse Control, Harare, Zimbabwe; National Institute of Allergy and Infectious Diseases, United States of America

## Abstract

**Background:**

In the savannahs of East and Southern Africa, tsetse flies (*Glossina* spp.) transmit *Trypanosoma brucei rhodesiense* which causes Rhodesian sleeping sickness, the zoonotic form of human African trypanosomiasis. The flies feed mainly on wild and domestic animals and are usually repelled by humans. However, this innate aversion to humans can be undermined by environmental stresses on tsetse populations, so increasing disease risk. To monitor changes in risk, we need traps designed specifically to quantify the responsiveness of savannah tsetse to humans, but the traps currently available are designed to simulate other hosts.

**Methodology/Principal Findings:**

In Zimbabwe, two approaches were made towards developing a man-like trap for savannah tsetse: either modifying an ox-like trap or creating new designs. Tsetse catches from a standard ox-like trap used with and without artificial ox odor were reduced by two men standing nearby, by an average of 34% for *Glossina morsitans morsitans* and 56% for *G. pallidipes*, thus giving catches more like those made by hand-nets from men. Sampling by electrocuting devices suggested that the men stopped flies arriving near the trap and discouraged trap-entering responses. Most of human repellence was olfactory, as evidenced by the reduction in catches when the trap was used with the odor of hidden men. Geranyl acetone, known to occur in human odor, and dispensed at 0.2 mg/h, was about as repellent as human odor but not as powerfully repellent as wood smoke. New traps looking and smelling like men gave catches like those from men.

**Conclusion/Significance:**

Catches from the completely new man-like traps seem too small to give reliable indices of human repellence. Better indications would be provided by comparing the catches of an ox-like trap either with or without artificial human odor. The chemistry and practical applications of the repellence of human odor and smoke deserve further study.

## Author Summary

In savannah areas of Africa the incidence of sleeping sickness is commonly low because the species of tsetse fly that spread the disease there feed mainly on wild and domestic animals, and are strongly repelled by humans. Environmental stresses can make the flies less responsive to the repellence, so threatening to increase greatly the disease risk. Man-like traps for tsetse could monitor the repellence, but the only traps available are ox-like. Hence, we tried two approaches to developing man-like traps: first, the modification of an existing ox-like trap, and second, the creation of entirely new traps that look and smell somewhat like people. The new traps caught very few tsetse, giving unreliable indices of disease risk. A better index is provided by monitoring the catches from an ox-like trap, to assess tsetse abundance, and comparing such catches with those from an ox-like trap provided with artificial human odor, to assess repellency. Geranyl acetone seems to be an important repellent in human odor, but it is not as effective as wood smoke. The chemistry and practical uses of repellents need further study.

## Introduction

Human African trypanosomiasis (HAT), also known as sleeping sickness, is caused by certain subspecies of *Trypanosoma brucei* and is transmitted to humans by tsetse flies (*Glossina* spp) [1]. Several thousand cases are recorded annually [1] but due to under-diagnosis and poor reporting the true number of cases is probably much greater [2]. Most cases are caused by *T. b. gambiense*, transmitted by those species of tsetse, such as *G. fuscipes* Newstead and *G. palpalis* Robineau-Desvoidy that inhabit riverine woodland in West and Central Africa and which are strongly attracted to humans [3], [4]. The species of tsetse that inhabit savannah, such as *G. morsitans* Westwood and *G. pallidipes* Austen, transmit *T. b. rhodesiense* and pose less of a threat because they feed mainly on wild and domestic animals and are repelled by people [5]. However, the efficacy with which savannah tsetse are repelled by humans varies according to season [6] and the abundance of normal hosts [7], [8], [9]. Hence, ecological shifts, perhaps associated with changes in land use and climate, could trigger marked increases in the incidence of HAT in savannah areas. For example, the increase in the apparent monthly risk of HAT as temperatures rise in certain seasons of a single year [6] suggests that rises in annual mean temperatures over several decades could enhance the yearly risk. In addition to the direct impact of HAT on human health, there is an economic danger – an upsurge in the few cases currently recorded annually from the large national parks in the savannahs of East and Southern Africa [10] might reduce the appeal and revenues of these important tourist destinations. Thus, for various reasons, it would be wise to monitor HAT risk to give timely warnings of the need for intervention.

While it is essential to continue monitoring the numbers of HAT cases reported [10], it must be recognized that such monitoring is retrospective and that records of new cases can take many months to filter through from the far-distant diagnostic centers used by tourists. Moreover, where the incidence is now very low and the diagnosis and reporting is inefficient, it might take several years to expose confidently that the disease risk is rising. For example, is an upsurge in the number of recorded cases due to a real increase in incidence or merely an improvement in diagnosis? Earlier and more reliable warnings might be produced if records of cases were supplemented by the type of risk index suggested for use with riverine tsetse [11]. That index involves: (i) trap catches of tsetse as indicators of population abundance, (ii) the proportion of humans in the identification of tsetse bloodmeals, and (iii) the proportion of tsetse infected with *T. brucei*. However, the bloodmeal identifications and infection studies are complex, costly and long-winded. This is especially so with the savannah tsetse for which the proportion of humans in diet [12] and the *T. brucei* infection rate of the flies [13], [14] are typically very low, so that the confident and timely assessment of any changes would require the examination of thousands of tsetse per month. If the use of traps for routine monitoring for HAT risk in savannah situations is to be practicable, it should involve something quicker, simpler and cheaper, even if less comprehensive. For this it would be useful to design a trapping system by which the mere counts of daily catches can indicate changes not only in tsetse abundance but also in the efficacy of human repellence. Such a system demands a trap that simulates a man.

Unfortunately in the present context, the aim in trap design has so far been to catch as many flies as possible, and so traps have been produced to simulate particularly attractive features of the environment. For example, the Wigwam trap represented refuges, such as a rot-hole in a tree, to which many tsetse go during the hot season [15]. More usually, however, traps were produced to simulate host animals that are attractive at all seasons. Thus the traps of Harris [7] and Morris [16] were made to appear like large and small herbivores, respectively. More recently, traps such as the Epsilon [17], were created for use with chemical attractants identified from ox odor [18]. Such traps are termed “ox-like” because they give catches like those from oxen, although the traps do not appear much like oxen to humans. No trap seems to have been designed specifically to simulate a man for savannah tsetse – hardly surprising with such flies since humans are so repellent.

In a purely scientific sense, the best means of producing a man-like trap would be to design a trap that duplicated all of the effective stimuli from men. However, while this approach is theoretically ideal it would require complex and costly equipment to quantify the whole sequence of responses to men, and to establish the degree to which various stimuli affect each separate response – in the same way that ox-like traps were produced by detailed study of responses to cattle [18]. It might be speedier and cheaper if a man-like trap could be produced from the existing designs of trap by merely adding or removing something simple, especially if this meant that the same basic trap could be set to operate in man-like or ox-like mode to suit the entomologist's varying requirements from day to day.

Fortunately, whatever approach one adopts, a useful first step might be to explore further the indication that the catches of *G. morsitans morsitans* and *G. pallidipes* from an ox-like trap were affected by having a man nearby [19]. That indication was produced from work performed only in cool dry season, and used an early precursor of the Epsilon trap baited with the best odor attractants then available, ie, carbon dioxide and acetone. It is necessary to determine whether the same phenomenon occurs in other seasons, using the Epsilon itself, with and without the odor baits currently recommended. Present work with *G. m. morsitans* and *G. pallidipes* in Zimbabwe investigated these matters, explored the stimuli responsible for the effect of men and tested several new traps, mainly using simple techniques that could be employed readily with other tsetse species elsewhere.

## Study Area and General Methods

All studies were performed in woodland about 1 km from Rekomitjie Research Station (16° 10′ S, 29° 25′ E, altitude 503 m) in the Mana Pools National Park of the Zambezi Valley, where *G. m. morsitans* and *G. pallidipes* occur. During the 53 years of research at Rekomitjie, no case of HAT has been found to be contracted there, despite the good diagnostic facilities of the station and the fact that the scientists and their staff are bitten in the course of their normal duties [6].

### Ethics

The procedures for sampling tsetse followed long-standing protocols practiced at Rekomitjie. All persons used as catchers or baits in the experiments were permanent pensionable employees of the Division of Tsetse and Trypanosomiasis Control, Government of Zimbabwe and given regular updates on the purpose and results of the studies. Before recruitment, the Division explains the nature of the work, the risks associated with tsetse, other disease vectors and wild animals, and warns of the social hardships attending life on a remote field station. Recruits sign a document indicating their informed consent to perform the work required. This document is held by the Division. All experiments were given ethical approval by the Division's Review Committee for Rekomitjie.

### Traps and odors

**Figure 1:**
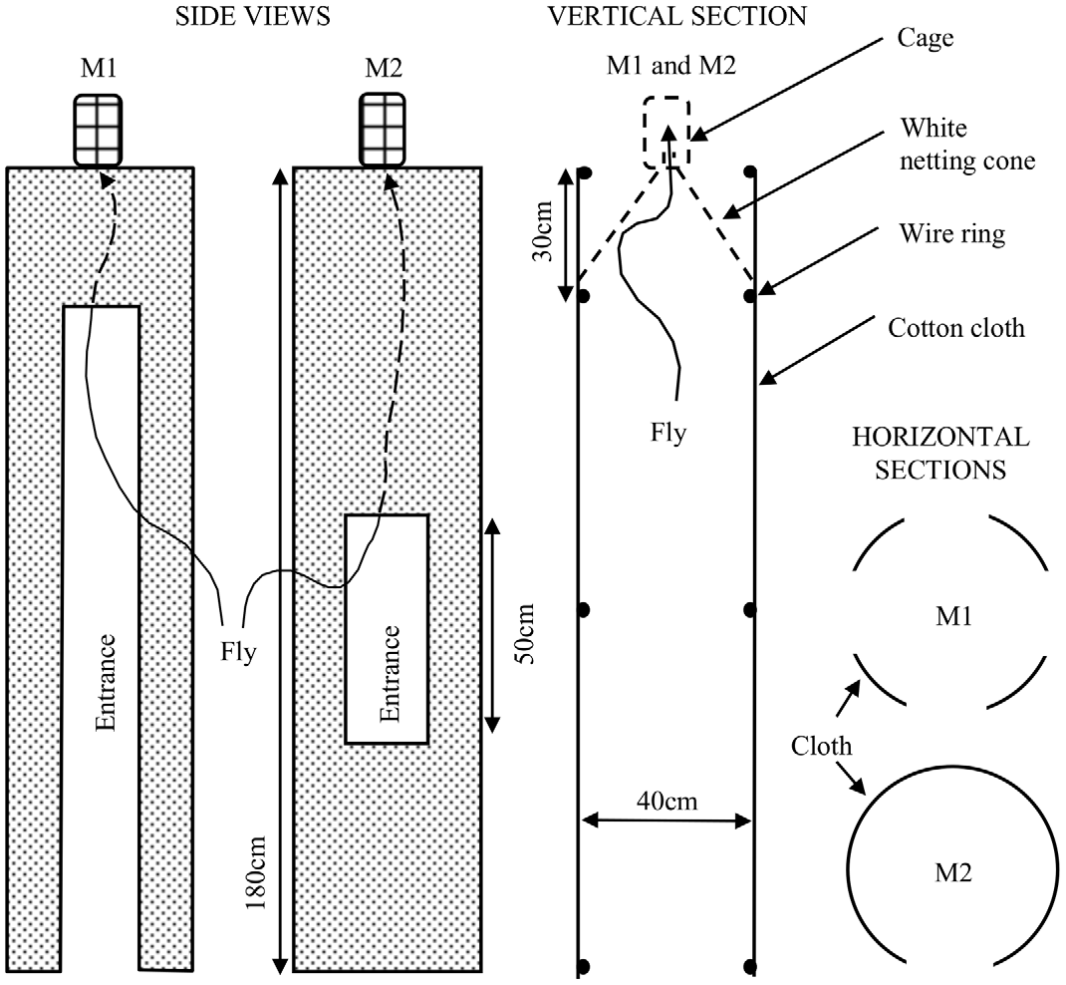
Structure of the M1 and M2 traps. Each was used as variants in which the cloth component was all black or all white.

**Figure 2:**
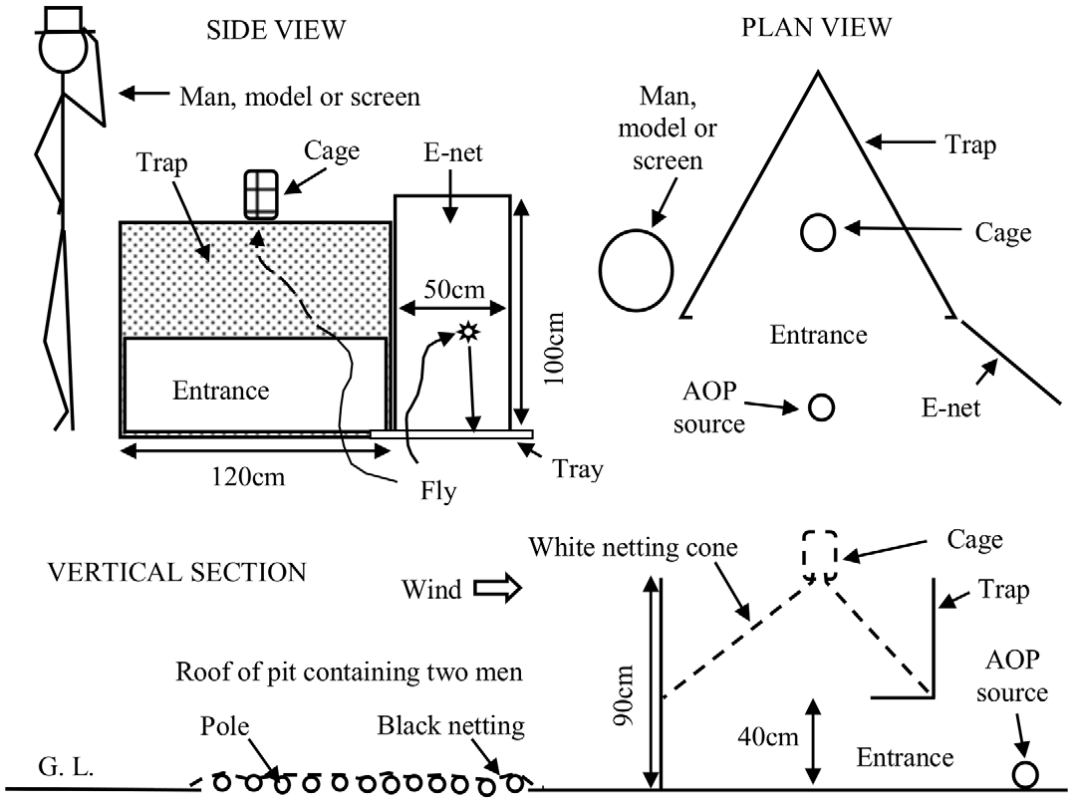
Location of various items used with Epsilon traps. When present, the men, models, screens and E-nets were paired, one on each side of the trap entrance, but only one of a pair is shown.

The standard trap was the Epsilon [17], 90 cm tall and 120 cm wide, made of Phthalogen blue cotton cloth. Various traps of more man-like shape, called M1 and M2 traps, were made of either black or white cotton cloth (Fig. 1). Traps were sometimes baited with simulated ox odor, termed AOP, comprising 100 mg/h acetone, 0.5 mg/h 1-octen-3-ol, 0.1 mg/h 3*n*-propyl phenol and 1.0 mg/h of 4-methyl phenol, dispensed according to [20]. Other wind-borne materials included smoke from a small fire made from one or two smoldering logs of *Colophospermum mopane*, about 5 cm in diameter and 25 cm long, placed 30 cm downwind of the trap on a circular, rusted steel tray 45 cm in diameter. Such smoke is known to be potently repellent [21]. Geranyl acetone and 6-methyl-5-hepten-2-one were tested since they have been identified in human odor and appear to repel mosquitoes [22]. These chemicals were dispensed from polyethylene sachets giving doses of about 0.2 mg/h of geranyl acetone and 2 mg/h of 6-methyl-5-hepten-2-one at 22°C. The sachets were 5 cm square, with a wall thickness of 120 µ, and were placed next to the AOP dispensers (Fig. 2).

### Humans and models

The human subjects were African males, in cotton overalls that were mono-chrome, either green, black or white, or striped with black and white – the stripes being produced by sewing black cotton strips, 5 cm wide, to white overalls, leaving white bands of similar width. The men were used in pairs, with a man about 50 cm from each side of the trap (Fig. 2). Unless stated otherwise, the men were in green overalls and standing. Human odor in the absence of visual stimuli from men was produced by two men sitting in a pit, 110 cm deep by 140 cm wide by 110 cm long, 50 cm upwind of the trap (Fig. 2). The pit was roofed at ground level with poles 5 cm in diameter, spaced about 5 cm apart and covered with black netting. This allowed odor to disperse from the pit, but prevented access by the few tsetse that approached the roof. The visual stimuli from men were duplicated by oblongs of black or white cotton cloth, 180 cm tall and 40 cm wide, or by models consisting of overalls stuffed with straw, using poles as internal supports, and molded bundles of straw to represent head and hands.

### Electrocuting nets and hand-nets

In some studies the tsetse flying near men and traps were caught by electrocuting nets (E-nets) [23], 100 cm tall and 50 cm wide, consisting of a grid of fine black copper wires placed over the surface of fine black netting. Flies electrocuted on collision with the E-nets fell to be retained in trays consisting of corrugated fibre-glass sheets placed on the ground and coated with sticky polybutene. When used with traps the E-nets were placed one at each of the two corners near the trap entrance (Fig. 2). When employed with two men standing alone, an E-net was beside each man. In some other work tsetse alighting on men or on the outside of traps were caught using hand-nets. However, unless stated otherwise, the hand-nets and E-nets were not used, ie, catches refer only to flies that entered the traps to be retained in the trap cage.

### Statistics

In a number of separate experiments performed in the last three hours before sunset, from August 2010 to April 2012, baits of different type were allotted to a series of randomized Latin squares of baits×days×sites, with the sites being about 200 m apart in woodland. The various baits used in each experiment were chosen primarily to elucidate trap design. However, in all experiments two of the baits were always an Epsilon trap alone and such a trap with two men nearby, so giving indications of any change in the effect of the men from one experiment to another. For analysis, the daily catches were transformed to log(n+1), to normalize their distributions. Reporting of the results focuses on the detransformed mean catches – an example of the transformed details being given in Table S1. The term “significant” implies significant differences between transformed means at the 0.05 level of probability. If an F test indicated significant heterogeneity in the set of transformed means of baits, the differences mainly responsible for the effect were identified via the least significant difference, ie, standard error ×1.4142×*t*.

## Experiments and Results

### Expt 1: Epsilon traps with men and AOP

Traps were used with and without AOP and with and without real men. The results (Table 1) showed that in the presence or absence of men the AOP increased catches significantly, by 3–9 times, consistent with the known potency of this odor [18]. In the presence of men, the catches of female *G. m. morsitans* and male and female *G. pallidipes* were reduced, usually by about half, with the effect being significant in several cases. By contrast, the catches of male *G. m. morsitans* seemed if anything to increase in the presence of the men, although the effect was not significant. Overall the effect of men with the present Epsilon trap was roughly compatible with the effect found with the prototype of this trap used in earlier work [19], which showed, for example, that the presence of men reduced the catches of female *G. pallidipes* by an average of 54%.

**Table 1:** Catches from an Epsilon trap with and without men and with and without AOP, in Expt 1.

Treatment		*G. m. morsitans*		*G. pallidies*	
		Males	Females	Males	Females
Without AOP	Trap alone	0.86 a	4.15 ac	7.26 a	40.95 a
	Trap+men	1.20 a	2.37 a	3.17 b	13.91 b
With AOP	Trap alone	5.73 b	13.77 b	32.90 c	118.57 c
	Trap+men	6.01 b	6.30 c	27.38 c	64.69 d

Detransformed mean daily catches of 12 daily replicates in Aug 2010. Means not associated with the same letter differ at the 0.05 level of probability.

### Expts 2 and 3: Epsilon traps and men with hand-nets

To start explaining the effect of men with the traps and to compare the catches from traps and men alone, studies were made with: (i) a trap alone, (ii) a trap with men not using hand-nets, (iii) a trap with men who used hand-nets to catch tsetse from themselves and the outside of the trap, and (iv) men alone using hand-nets. In Expt 2, in April 2011, the baits were operated with AOP, but in Expt 3, in May 2011, AOP was not used. In the results presented in Table 2, the catches from the traps+men with hand-nets refer to the combined catch from the hand-nets and the trap cage.

**Table 2:** Catches from Epsilon traps with and without men, and from men alone, in Expts 2 and 3.

Expt and treatment	*G. m. morsitans*		*G. pallidies*	
	Males	Females	Males	Females
**Expt 2. Apr 2011, 16 replicates, with AOP**				
Trap alone	2.19 a	4.23 a	13.38 a	55.44 a
Trap+men	1.56 ac	2.24 ac	5.52 b	14.83 b
Trap+men with hand-nets	4.79 b	1.79 c	4.52 b	12.18 b
Men alone with hand-nets	0.63 c	0.04 d	0.04 c	0.12 c
**Expt 3. May 2011, 16 replicates, without AOP**				
Trap alone	0.30 a	0.89 ac	1.37 a	7.05 a
Trap+men	1.08 b	0.31 ad	0.69 a	1.18 b
Trap+men with hand-nets	2.11 b	0.99 bc	0.74 a	1.45 b
Men alone with hand-nets	0.59 a	0.09 d	0.00 c	0.00 c

Detransformed mean daily catches. Means not associated with the same letter differ at the 0.05 level of probability. In all treatments involving a trap, catches were made in the trap cage. When the men were alone they used hand-nets to catch tsetse from themselves, and when with a trap, they either used no hand-nets (Trap+men) or made hand-net catches from themselves and the outside of the trap (Trap+men with hand-nets). In the latter case, the catches from the trap cage were pooled with those by hand-nets before analysis.

In accord with the findings of Expt 1, the catches in Expt 2 with AOP were around 5–10 times greater than in Expt 3 without AOP (Table 2). However, certain trends were evident in the presence and absence of AOP, even if the trends were not always significant. First, although the catches of male *G. m. morsitans* from the trap alone did not differ grossly or consistently from the hand-net catches from men alone, the trap alone gave ten to several hundred times more female *G. m. morsitans* and male and female *G. pallidipes* than the hand-net catches from men alone, thus confirming that catches of Epsilon traps are poor indications of the numbers of tsetse that approach humans. Second, while the supplementary hand-net catching seemed to double the numbers of male *G. m. morsitans* caught from the trap+men, there was no such marked effect with the other tsetse.

Records were made of where the flies were caught when the trap+men was used with hand-nets. The data (Table 3) show that the number of flies caught inside the trap, ie, in the trap's cage, relative to the total hand-net catches made outside, ie, from the men or the trap surface, were many times greater for females than for males, and for *G. pallidipes* than for *G. m. morsitans*. This accords with other evidence that the propensity to enter traps is greatest with females and with *G. pallidipes* [24]. Furthermore, for both sexes of both species the proportion of the catch made in the cage increased several-fold in the presence of AOP, consistent with other work indicating that AOP encourages trap-entering responses [25].

**Table 3:** Distribution of total catches at a trap + men with hand-nets, in Expts 2 and 3.

Expt and bait	Method	*G. m. morsitans*		*G. pallidies*	
		Males	Females	Males	Females
**Expt 2. Apr 2011, 16 replicates, with AOP**					
Trap	Cage	48	96	253	1011
Trap	Hand-nets, on trap	77	9	15	4
Men	Hand-nets, on men	3	1	1	0
	(Cage/hand-nets)	(0.6)	(9.6)	(15.8)	(252.8)
**Expt 3. May 2011, 16 replicates, without AOP**					
Trap	Cage	6	20	27	128
Trap	Hand-nets, on trap	27	6	3	5
Men	Hand-nets, on men	3	4	2	2
	(Cage/hand-nets)	(0.2)	(2.0)	(5.4)	(18.3)

Catches were made in the trap cage, and by hand-nets from the men and from the outside of the trap. Cage/hand-nets is the catch from the trap cage as a proportion of the total hand-net catches, ie, from the men and the outside of the trap.

More intriguingly, however, the data (Table 3) show that with males and females of both species the hand-net catches from the men were lower than the hand-net catches from the trap. Moreover, if the hand-net catches from the trap are added to the trap cage, to give the overall catches from the trap, it emerges that the trap catches were many times greater than from the nearby men, with the effect being least for male *G. morsitans* and greatest for female *G. pallidpes*. It appears, therefore, that the distribution of tsetse around the combination of the Epsilon trap+men parallels the distribution around an ox+men observed in other studies [6], [8], in which by far the most flies were caught from the ox. The extents to which the presence of men reduced the catches of each sex and species from the trap correspond roughly with the reductions in catches from oxen when men are nearby [5]. These results are consistent with the view that the Epsilon trap is much more ox-like than man-like.

### Expt 4: Epsilon traps and men with E-nets

The catches in the above two experiments indicate the overall result of: (i) the numbers of flies attracted to the vicinity of the baits and (ii) the way that the flies behaved after arrival, ie, alighting on the baits or entering the trap. Expt 4 was performed to focus more on the number of flies that visited the baits, using E-nets with: (i) a trap alone, (ii) trap with men, (iii) men alone and (iv) no bait, ie, an E-net alone. AOP was used in all four treatments to ensure greater catches. The results (Table 4) show that the trap alone caught about 10–30 times more tsetse than the men – indeed, the men alone caught about as many flies as when no bait was used, suggesting that the men were hardly attractive at all. The presence of the men seemed to reduce the catches of the trap by about half, although this apparent effect was not as great as in Expts 1–3, and was not significant.

**Table 4:** Catches by E-nets used with and without an Epsilon trap and with and without men, in Expt 4.

Treatment	*G. m. morsitans*		*G. pallidies*	
	Males	Females	Males	Females
Trap+E-nets	6.86 a	15.67 a	34.27 a	138.77 a
Trap+men+E-nets	5.26 a	8.81 a	15.68 a	59.56 a
Men+E-nets	0.97 b	0.83 b	1.02 b	4.64 b
E-nets alone	0.76 b	0.53 b	1.86 b	5.01 b

Detransformed mean daily catches of 10 daily replicates in September 2011. Means not associated with the same letter differ at the 0.05 level of probability. All treatments included AOP.

It is pertinent, however, to examine the distribution of catches between the trap cage and the E-nets (Table 5). Whether the men were present or absent, relatively few of the flies were caught in the trap cage, suggesting that most flies flew round the trap before attempting to enter it. Nevertheless, the proportion of *G. pallidipes* that flew straight into the entrance was reduced significantly, by about half, when men were present (P<0.01 for each sex, by chi-squared). This reduction does not seem to be an indirect effect due merely to the flies being diverted from the trap to the men, since catches from men alone were small (Table 4). Moreover, *G. m. morsitans*, the species that might have been expected to show most clearly any such diversion, seemed to show little or none, in that the catch in the cage, as against at the E-nets, was hardly affected by the presence of the men (P>0.05 for each sex). Hence, it appears the men had a direct impact on trap-entering responses of *G. pallidipes*.

**Table 5:** Distribution of total catches made by the trap cage and E-nets, at a trap alone or a trap with men, in Expt 4.

Bait and catching method		*G. m. morsitans*		*G. pallidies*	
		Males	Females	Males	Females
Trap alone	Cage	5	18	52	146
	E-nets	74	198	413	1711
	(Cage/E-nets)	(0.07)	(0.09)	(0.13)	(0.09)
Trap+men	Cage	5	10	8	34
	E-nets	69	137	178	707
	(Cage/E-nets)	(0.07)	(0.07)	(0.04)	(0.05)

AOP was used with both baits. Cage/E-nets is the cage catch as a proportion of the E-nets catch.

In Expt 4 the cage catch relative to that from the E-nets (Table 5) was very much lower than the cage catch relative to flies caught by hand-nets in Expts 2 and 3 (Table 3). This is because E-nets catch tsetse much more efficiently than hand-nets [5], so that with the hand-nets the flies have less chance of being caught before entering.

Combining the indications of Expts 2–4, it appears that the differences between the catches from men and Epsilon traps have two causes: (i) men attract fewer flies and (ii) men induce distinctive responses at close range. These matters ensure that when men are beside the trap the magnitude and composition of catches from it approach more closely the catches from men alone. The next set of experiments explored the stimuli associated with this effect.

### Expts 5–10: men and man-like stimuli with Epsilon traps

The results of Expt 5 (Table 6) showed that the reduction in trap catches due to nearby men was much the same whatever the color and pattern of the overalls the men wore. Expt 6 (Table 6) indicated that there was some effect according to whether the men were standing upright as against sitting or lying flat, and that the effect varied according to the sex and species of tsetse. However, the over-riding indication was that for those tsetse most affected by human presence, ie, female *G. m. morsitans* and both sexes of *G. pallidipes*, the men in any position produced a marked and significant reduction of catches in almost all cases. Expts 7 and 8 (Table 6) indicated that whether AOP was absent, as in Expt 7, or present, as in Expt 8, the type of effect due to the presence of real men could be reproduced *qualitatively* either by the odorless model men alone or by human odor alone. However, in Expt 8 where the large catches of *G. pallidipes* offer the most reliable indications, it appears that the repellent effect of human odor alone was *quantitatively* greater than that of the models alone, and produced effects almost as marked as the combination of the models and human odor. The latter combination was roughly as effective as the real men, suggesting that the occasional movements and conversation sounds from the real men had little or no impact.

**Table 6:** Catches from various baits in Expts 5–10.

Expt and treatment	*G. m. morsitans*		*G. pallidies*	
	Males	Females	Males	Females
**Expt 5. Sep 2010, 16 replicates, without AOP**				
Trap alone	0.69 a	5.48 a	5.67 a	46.92 a
Trap+upright men	0.88 a	1.36 c	5.02 ab	15.57 b
Trap+sitting men	1.32 a	3.19 b	3.16 b	10.75 c
Trap+flat men	1.31 a	1.57 c	1.66 d	9.66 c
**Expt 6. Oct 2010, 18 replicates, without AOP**				
Trap alone	0.77 a	1.20 a	4.00 a	8.86 a
Trap+men, green overalls	1.55 a	0.52 a	3.57 a	5.70 b
Trap+men, black overalls	1.12 a	1.14 a	2.94 ab	3.69 c
Trap+men, white overalls	1.14 a	0.68 a	1.97 b	4.12 bc
Trap+men, H striped overalls^1^	0.90 a	0.61 a	2.01 b	4.16 bc
Trap+men, V striped overalls^1^	1.42 a	1.10 a	1.89 b	4.17 bc
**Expt 7. Feb 2011, 15 replicates, without AOP**				
Trap alone	0.42 abc	0.49 a	1.19 a	2.46 a
Trap+men	0.93 a	0.69 a	1.04 ab	1.06 b
Trap+model men	0.05 c	0.38 ab	0.51 bc	1.73 ab
Trap+men odour	0.62 ab	0.10 b	0.42 c	1.25 b
Trap+model men+men odour	0.27 bc	0.26 b	0.85 bc	1.31 ab
**Expt 8. Mar 2011, 15 replicates, with AOP**				
Trap alone	2.07 a	5.21 a	13.03 a	34.18 a
Trap+men	1.49 a	1.34 b	4.59 bd	8.09 bd
Trap+model men	2.66 a	3.61 a	9.16 ac	23.44 c
Trap+men odour	1.47 a	1.42 b	5.92 bc	8.66 b
Trap+model men+men odour	1.79 a	1.17 b	3.23 d	5.21 d
**Expt 9. Jun 2011, 16 replicates, with AOP**				
Trap alone	1.17 a	3.59 a	13.86 a	48.28 a
Trap+men	1.04 a	1.33 b	5.64 b	14.78 b
Trap+white vertical oblong	0.71 a	1.38 b	5.60 b	19.37 b
Trap+black vertical oblong	0.94 a	0.58 b	4.48 b	13.32 b
**Expt 10. Feb-Apr 2012, 36 replicates, with AOP**				
Trap alone	0.34 a	0.56 b	3.85 a	8.23 a
Trap+men	0.27 a	0.34 bc	1.83 b	2.76 b
Trap+GA odour^2^	0.17 ab	0.13 cd	0.53 c	0.70 c
Trap+6M odour^2^	0.32 a	0.91 a	3.91 a	9.51 a
Trap+GA+6M odours^2^	0.04 bc	0.15 cd	0.77 c	0.78 c
Trap+smoke	0.00 c	0.02 d	0.02 d	0.16 d

Detransformed mean daily catches. Means not associated with the same letter differ at the 0.05 level of probability. Unless stated otherwise, men and model men were upright in green overalls.

1 H = horizontal, V = vertical.

2 GA = geranyl acetone, 6M = 6-methyl-5-hepten-2-one.

Expt 9 (Table 6) suggested that the visual effect of men could be produced by simplified models consisting of the black or white vertical oblongs of cloth. Expt 10 showed that the repellence of human odor could be duplicated by geranyl acetone in the presence or absence of 6-methyl-hepten-2-one. However, geranyl acetone was not as powerfully repellent as smoke. There was no evidence that 6-methyl-hepten-2-one was repellent. Indeed, it seemed mildly attractive for female *G. m. morsitans* – although studies with larger catch sizes would be required to elucidate the point convincingly.

### Expts 11 and 12: Epsilon traps and M traps

The M traps used with natural human odor represented the best available simulations of the combined visual and olfactory stimuli from men. To assess the performance of such traps, an experiment of 16 replicates in July 2011 compared: (i) black M1, (ii) white M1, (iii) Epsilon trap alone and (iv) Epsilon trap with standing men. All traps had AOP, but only the M1 traps had human odor. The next experiment, of 12 replicates in August 2011, was the same except that it substituted the M2 traps for M1 traps.

In both of these experiments the daily catches from the Epsilon trap alone were, as expected from Expts 1–10, large, and the catches from the Epsilon trap with men were about half as great. However, the catches from any of the M-traps were always exceedingly low, and on almost all days were nil. On average the total catches from the M traps with human odor were 0.1% of those from the Epsilon trap, and such few flies as were caught from the M traps were mostly *G. m. morsitans*, ie, four out of five (Table 7).

**Table 7:** Total catches from an Epsilon trap with and without men and from M traps with human odor, in Expts 11 and 12 combined.

Bait	*G. m. morsitans*		*G. pallidipes*	
	Males	Females	Males	Females
Epsilon trap alone	52	162	455	1633
Epsilon trap+men	59	67	247	655
Black M traps+human odor	1	3	0	0
White M traps+human odor	0	0	0	1

Catches refer to a total of 28 daily replicates in Jul–Aug 2011. AOP was used with all baits. Data for the M traps of each color involve 16 replicates with the M1 form in Expt 11 combined with 12 replicates with the M2 form in Expt 12.

To increase the sample size from the black and white M2 traps, each was operated for 50 days in August to November 2011, with AOP, as in Expts 11 and 12, but without human odor. The total catch from the black M2 was eight *G. m. morsitans* and 16 *G. pallidipes*, as against nil and 12, respectively, from the white M2. The proportion of *G. pallidipes* was high in both catches, presumably because human odor was absent.

### Monthly collation

The present work was not organized primarily to expose any monthly change in the repellence of men. Indeed, the work was imperfect for such an aim since the sites and men were not always exactly the same from month to month, and AOP was used in some experiments but not others. Nevertheless, to gauge crudely any monthly change in human repellence, attention was focused on data for the effect of men next to an Epsilon trap in the most repeated type of experiment, ie, that involving flies being caught only in the trap cage. The total catches of males and females in each calendar month were pooled for all experiments of that month, and the monthly total for the trap+men was expressed as a percent of the total from the trap alone. The lower this percent the greater the apparent repellence of men, and hence the lower the proportion of the tsetse population that might bite humans. This simple means of reporting the data is useful in showing the way that results might be viewed by field staff of monitoring agencies.

The percents (Table 8) show that repellence was evident for *G. pallidipes* in all months. For *G. m. morsitans* the apparent repellence was a little less consistent, perhaps because the small sample sizes in some months gave unreliable indications. However, in each month the percent for *G. m. morsitans* was greater than for *G. pallidipes*, and was 1.5 times greater in all months combined. For *G. pallidipes* the percent ranged between 31 and 51% during the relatively cool period from April to September, compared to 77% in the very hot weather of October when enhanced proportions of the tsetse population are known to be heat stressed [26], young [27] and with low food reserves [9], and hence less selective in responses to hosts. However, temperature seems not be the only important matter since in the warm wet period of February–March the percent was high in February (59%) and declined particularly sharply on going to March (26%). This abrupt change was evident in both years studied, ie, in 2011 the percents for were 47% (N = 95) for February and 25% (975) for March, and in 2012 the percents were 68% (134) and 31% (236), respectively.

**Table 8:** Catches from an Epsilon trap alone as percent of the catch of the trap with men, in various months.

Month	Max °C	Rain mm	*G. m. morsitans*		*G. pallidies*	
			%	N	%	N
February	32.9	85.3	131.3	32	59.4	229
March	32.6	89.3	46.1	167	26.3	1211
April	31.9	47.2	55.7	144	35.7	1264
May	30.9	0.0	134.6	26	31.0	155
June	29.0	0.1	52.9	104	37.3	1341
July	27.3	0.0	52.8	89	50.9	737
August	29.9	0.0	71.0	521	50.4	4016
September	34.3	0.0	44.3	131	38.5	955
October	38.3	0.0	122.4	58	76.7	339
All months combined			65.6	1272	43.5	10247

Data refer to the pooled untransformed catches for all experiments performed in the month, irrespective of year. The maximum temperature is the average of daily values, and the rain is the mean monthly total. N is the catch from the trap alone, % is the catch of the trap with men, as a percent of N. Men were upright and in green overalls.

## Discussion

Our results showed that the presence of men beside Epsilon traps reduced the catches of the traps by about half for female *G. m. morsitans* and male and female *G. pallidipes*. The effect was less marked for male *G. m. morsitans*, and there is an indication that it was also less marked at the start of the very hot season and in part of the warm wet season, as against the cooler period in mid year. The catch reductions were due to the men preventing many flies from arriving near the traps and also to weakening of trap-entering responses. The effective stimuli from men were partly visual, but human odor seemed more important. Very few tsetse were caught from the M traps, which simulated the upright form of men and were used with human odor.

Taken at face value, the small catches from the M-traps baited with human odor, and the predominance of *G. m. morsitans* in the catch, ie, four out of five, suggest that these traps delivered samples that simulated well the catches of tsetse alighting on humans. Much more work, involving greater sample sizes, would be required to establish this point cogently. However, even if it were shown that sampling by any of the M-traps could properly substitute for catches from men, it would be unsatisfactory to standardize on traps that yield so few flies each day. The efficacy of humans would be exposed more clearly by systems that deliver larger catches that can be given statistical weight more readily. In this regard, the Epsilon trap with human stimuli would be more appropriate. Admittedly, the catch from the Epsilon with such stimuli was hardly comparable with those from stationary men, but adding the stimuli exposed human repellence convincingly, so that a material change in human repellence could be expected to be exposable also.

Hence, it seems that a very simple means of monitoring some of the important factors in HAT risk associated with savannah tsetse would be to operate two sorts of trap simultaneously: (i) a standard trap, such as the Epsilon, used alone, and (ii) such a trap used with artificial human odor. A cross-over design, involving just one example of each sort of trap alternated between daily between two sites, would produce the type of data that appear pertinent (Table 8), and it would probably be adequate to operate no more than one such study per 100–1000 km^2^. The catch from the standard trap would provide an index of the overall abundance of tsetse, and the extent to which catches are reduced by artificial human odor would show the degree of human repellence. To maximize the catch at both trapping systems it would be appropriate to use AOP with those species for which such attractants are effective. While the present data hardly prove that human repellence is exactly the same in the presence and absence of AOP, it is shown that AOP does not prevent the repellence from being detected clearly.

Unfortunately, any change in the efficacy of human repellence with the stationary Epsilon trap would not necessarily be identical to that occurring with mobile baits, it being known that mobile and stationary baits attract distinctive samples [5], [28]. However, the repellence of humans with mobile baits seems no less than with stationary ones, and much of the repellence with mobile baits is known to be olfactory [5], [29]. Thus, it seems that the trapping system proposed to assess any changed efficacy of human odor would pertain to risks experienced by mobile and stationary people.

A fuller appraisal of the value of the dual trapping system and the ways in which it might be improved must await the system being tried with other tsetse species and in other places, especially where the incidence of HAT is higher than at Rekomitjie. For example, it would be instructive to explore its use with savannah tsetse such as *G. swynnertoni* at Serengeti in Tanzania. Moreover, while there is no evidence that riverine tsetse are repelled by humans, it would be intriguing to see what happens when humans stand next to traps for such flies. Meanwhile, present data seem to offer a benchmark for the type of results associated with human repellence at or near its maximum. They also exemplify, in the following two matters, the sorts of thing that can be shown by the trapping system as it now stands, and emphasize some caveats.

First, since the reduction of trap catches due to nearby men was less for *G. m. morsitans* than for *G. pallidipes* it would appear, in the absence of any other considerations, that *G. m. morsitans* is the more important vector of HAT. The fact that *G. m. morsitans* is more available than *G. pallidipes* to mobile men [5], [6] would further increase the relative importance of *G. m. morsitans*, and accords with the fact that this species forms the majority of tsetse probing men at Rekomitjie [6]. Nevertheless, a high proportion of the few *G. pallidipes* that do feed on men are old enough to harbor mature infections of *T. brucei* [6], [30]. Thus, while *G. m. morsitans* seems to be the main vector, *G. pallidipes* cannot be ignored.

Second, although the present evidence for seasonal effects on the repellence of humans is crude, it is consistent with the observations that the age structure and nutritional state of tsetse populations change seasonally [6], [9], [27] and that the repellence of men is least with very young flies [5], [28] and when the food reserves of tsetse are low [5], [6]. For example, the repellence measured in the current study seemed weakest in the very hot weather of October, when the nutritional status of tsetse is poor [9] and the proportion of very young flies in the population rises markedly to its maximum annual level [27], according with the fact that the readiness to accept human hosts appears to be greatest at that time [6]. However, since young infected flies are not yet able to transmit their infection, an enhanced acceptance of humans at times when many flies are young need not necessarily mean a high risk of transmission. In any event, the interplay of season and the age structure, nutritional state and abundance of tsetse as factors in disease risk could be particularly important and complex at the interface of game parks and agricultural areas, especially since tsetse can diffuse between such habitats [31]. This might mean, for example, that old potentially-infective tsetse entering the farming area could starve and become highly responsive to men, so increasing the disease risk there, even if tsetse do not live long in agricultural situations. Hence, it could be particularly instructive to apply the dual trapping system and age studies across interfaces at various seasons.

Putting all of the above considerations together, it seems that in attempting to expose any change in HAT risk it would be safest to compare the dual trapping data of one month with not only those of the preceding month, but also with the data for the corresponding month in the previous year. Even then, any apparent reduction in repellence should be taken merely as an early warning that HAT risk might be increasing and that fuller studies are then required, covering particularly the age of tsetse. Furthermore, the proposed trapping system can monitor only the disease risk in the normal woodland habitat of the flies, whereas many of the flies probing men in the hot dry season do so in buildings [6]. Hence, it would be wise to supplement the trap data with occasional surveys of the tsetse in buildings, and to assess the extent to which trap catches outside of buildings can be used as indices of risk inside at different seasons. This business at Rekomitjie is to be addressed by a subsequent paper.

Regarding the type of artificial odor to use with man-like traps, geranyl acetone at the doses currently used seems as effective as human odor. Moreover, the fact that geranyl acetone occurs on the skin [22] accords with the finding that the repellent odor from humans emanates from the body surface [32], although the dose of geranyl acetone in present studies appears greater than that occurring in natural odor from people [22]. Thus, it is appropriate to see what other chemicals from humans, at what dose, can account for repellence. Moreover, it might be useful to identify the exceeding potent repellents present in smoke. Such repellents might not be strictly pertinent to simulating human odor as such, but it would be instructive to see whether factors influencing the repellence of human odor affect also the repellence of other odors in the human environment, and whether the response to a combination of artificial human odor and smoke chemicals would be a more sensitive yard-stick for exposing any changes in responsiveness to humans. Could a super-repellent mix provide added protection to people?

Given that human repellence seems to be the greatest thing preventing outbreaks of Rhodesian sleeping sickness in savannah areas, our understanding, monitoring and possible enhancement of the repellence are some of the most neglected aspects of this disease.

## Supporting Information

Table S1 **Transformed data for catches of female** ***G. pallidipes*** **in Expt 1, to compare an Epsilon trap with and without men, in the presence and absence of AOP.** The experiment involved three consecutive Latin squares, each of four baits×four sites×four days, making a total of 12 daily replicates with each treatment. Analysis of variance of the daily catches removed the effects of baits, sites and days, but only the bait effects are shown here. Since an F test indicated significant heterogeneity (P<0.05) among the bait means, the standard deviation (SD), standard error (SE) and the least significant difference between means (LSD) were calculated. Means not associated with the same letter differ significantly. In some other data sets, where F indicated no significant heterogeneity (P>0.05), **a** was placed next to all means of the set.
(DOC)
