# Supplementary material for: Towards an Early Warning System for Rhodesian Sleeping Sickness in Savannah Areas: Man-Like Traps for Tsetse Flies
Source: PLoS Negl Trop Dis. 2012 Dec 27;6(12):e1978. doi: 10.1371/journal.pntd.0001978 (PMC3531518; doi:10.1371/journal.pntd.0001978)
Supplement: Table S1 — Transformed data for catches of female G. pallidipes in Expt 1, to compare an Epsilon trap with and without men, in the presence and absence of AOP. The experiment involved three consecutive Latin squares, each of four baits×four sites×four days, making a total of 12 daily replicates with each treatment. Analysis of variance of the daily catches removed the effects of baits, sites and days, but only the bait effects are shown here. Since an F test indicated significant heterogeneity (P<0.05) among the bait means, the standard deviation (SD), standard error (SE) and the least significant difference between means (LSD) were calculated. Means not associated with the same letter differ significantly. In some other data sets, where F indicated no significant heterogeneity (P>0.05), a was placed next to all means of the set. (DOC) [file pntd.0001978.s001.doc]

**Table S1.** Transformed data for catches of female *G. pallidipes* in Expt 1, to compare an Epsilon trap with and without men, in the presence and absence of AOP.

| Statistic | Bait or item | | Value |
| --- | --- | --- | --- |
| Mean catch | Without AOP | Trap alone | 1.622716 a |
|  |  | Trap + men | 1.173571 b |
|  | With AOP | Trap alone | 2.07762 c |
|  |  | Trap + men | 1.817479 d |
| Variance (df) | Baits (3) | | 1.746195 |
|  | Sites (3) | | 0.006425 |
|  | Days (11) | | 0.186733 |
|  | Residual (30) | | 0.025738 |
|  | Baits/residual = F (3/30) | | 67.84574 |
| For bait effects | P, from F | | 1.8E-13 |
|  | SD = residual variance^0.5 | | 0.160430 |
|  | SE = SD/12^0.5 | | 0.046312 |
|  | t, at P = 0.05, 30 df | | 2.042272 |
|  | LSD = SE x t x 2^0.5 | | 0.133759 |

The experiment involved three consecutive Latin squares, each of four baits x four sites x four days, making a total of 12 daily replicates with each treatment. Analysis of variance of the daily catches removed the effects of baits, sites and days, but only the bait effects are shown here. Since an F test indicated significant heterogeneity (P<0.05) among the bait means, the standard deviation (SD), standard error (SE) and the least significant difference between means (LSD) were calculated. Means not associated with the same letter differ significantly. In some other data sets, where F indicated no significant heterogeneity (P>0.05), **a** was placed next to all means of the set.
